# Obesity and cancer: the role of vitamin D

**DOI:** 10.1186/1471-2407-14-712

**Published:** 2014-09-25

**Authors:** Thurkaa Shanmugalingam, Danielle Crawley, Cecilia Bosco, Jennifer Melvin, Sabine Rohrmann, Simon Chowdhury, Lars Holmberg, Mieke Van Hemelrijck

**Affiliations:** Division of Cancer Studies, Cancer Epidemiology Group, King’s College London, School of Medicine, London, UK; Department of Oncology, Guy’s & St Thomas’ NHS Foundation Trust, London, UK; Division of Cancer Epidemiology, Institute of Social and Preventive Medicine, University of Zurich, Zurich, Switzerland; Regional Cancer Centre Uppsala/Orebro, Uppsala, Sweden; Department of Surgical Sciences, Uppsala University, Uppsala, Sweden

**Keywords:** Cancer, Obesity, Vitamin D

## Abstract

**Background:**

It is estimated that 20% of all cancer cases are caused by obesity. Vitamin D is thought to be one of the mechanisms underlying this association. This review aims to summarise the evidence for the mediating effect of vitamin D on the link between obesity and cancer.

**Methods:**

Three literature searches using PubMed and Embase were conducted to assess whether vitamin D plays an important role in the pathway between obesity and cancer: (1) obesity and cancer; (2) obesity and vitamin D; and (3) vitamin D and cancer. A systematic review was performed for (1) and (3), whereas a meta-analysis including random effects analyses was performed for (2).

**Results:**

(1) 32 meta-analyses on obesity and cancer were identified; the majority reported a positive association between obesity and risk of cancer. (2) Our meta-analysis included 12 original studies showing a pooled relative risk of 1.52 (95% CI: 1.33-1.73) for risk of vitamin D deficiency (<50 nmol/L) in obese people (body mass index >30 kg/m^2^). (3) 21 meta-analyses on circulating vitamin D levels and cancer risk were identified with different results for different types of cancer.

**Conclusion:**

There is consistent evidence for a link between obesity and cancer as well as obesity and low vitamin D. However, it seems like the significance of the mediating role of vitamin D in the biological pathways linking obesity and cancer is low. There is a need for a study including all three components while dealing with bias related to dietary supplements and vitamin D receptor polymorphisms.

**Electronic supplementary material:**

The online version of this article (doi:10.1186/1471-2407-14-712) contains supplementary material, which is available to authorized users.

## Background

Over recent decades, the increasing prevalence of obesity has been implicated in the risk of cancer incidence and mortality [1–3]. The link between obesity and cancer mortality is well-established [4, 5]. A prospective cohort study including >900,000 adults in the U.S, estimated that being overweight or obese could account for 14% of deaths from cancer in men and 20% in women [6]. In the UK, an estimated 17,294 excess cancer cases occurring in 2010, were due to overweight and obesity (5.5% of all cancers) [7]. However, the mechanisms that link excess body weight and carcinogenesis are not fully elucidated. Vitamin D is one of the factors suggested to play a role in this pathway [8], but the nature of this association is not fully understood [2]. The immune system and vitamin D receptor (VDR) are only two of the suggested mechanisms for a link between vitamin D and cancer which may also be connected to obesity [9–12].

**Figure 1:**
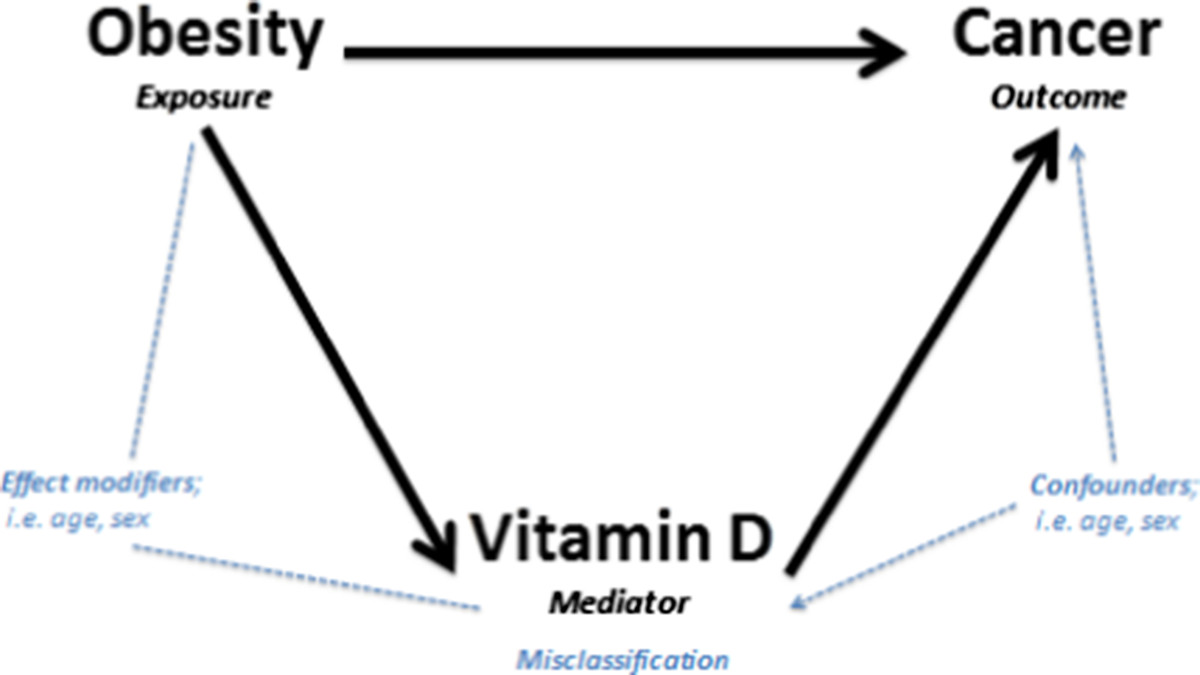
**Overview of vitamin D as a potential mediator for the association between obesity and cancer.** Abbreviations: TS, Thurkaa Shanmugalingam; DC, Danielle Crawley; BMI, body mass index.

To evaluate whether vitamin D explains how obesity affects cancer risk, one needs to assess if vitamin D is a mediator variable for the association between obesity (exposure) and cancer (outcome) [13, 14]. In a traditional epidemiological approach, mediation analyses would estimate the excess risk of obesity on cancer explained by vitamin D, by calculating the risk ratio for the association between obesity and cancer in a crude model, and a model adjusted for vitamin D [13]. To our knowledge, no mediation analyses have been published to date for this question, with the exception of one study focusing on breast cancer-specific mortality and one study estimating the attributable fraction of vitamin D in obese people [1, 15]. These studies were not set out as mediation analyses, but suggested that low vitamin D levels contribute to about 16 to 20% of the increased cancer incidence or mortality from breast cancer in overweight and obese patients [1, 15]. This is in contrast with findings from large cohort studies suggesting no association between vitamin D and breast cancer [16].We approached the issue of mediation by vitamin D with a literature review for each association with the question of whether vitamin D plays an important role in the pathway between obesity and cancer (Figure 1): (1) obesity and cancer; (2) obesity and vitamin D; and (3) vitamin D and cancer, while addressing some of the methodological issues. Many meta-analyses have been done for (1) and (3), but limited pooled results are available for (2). Hence, we performed a meta-analysis for the association between obesity and vitamin D.

## Methods

### Obesity and cancer

A comprehensive literature review of all published meta-analyses on the association between obesity and cancer was carried out. We used computerised search databases (PubMed search followed by an Embase search) to identify full text and abstracts focused on human subjects and published in English language within the last fifteen years. Searches were conducted both with and without MeSH terms for “obesity”, “cancer” and “meta-analysis”. This search was repeated for individual cancer types: “breast”, “colorectal”, “melanoma”, “oesophageal”, “liver”, “lung”, “ovarian”, “endometrial”, “prostate”, “pancreatic” and “kidney” cancer. Although lung cancer may not be the obvious cancer to investigate in the context of obesity [17, 18], some studies [19, 20] reported a positive association while others are inconclusive or conflicting. Hence, lung cancer was also included in this literature review.

### Obesity and vitamin D: a meta-analysis

#### Literature search strategy

We used computerised search databases (PubMed search followed by an Embase search) to identify full text and abstracts published within the last fifteen years, of English language and used human subjects. The searches were performed with and without MeSH terms for “vitamin D”, “25 hydroxyvitamin D”, “obesity”, and “body mass index”. We also included “grey literature” such as abstracts, letters, and articles presented at relevant conferences and meetings. All references of the selected articles were checked using hand searches.

#### Inclusion criteria

**Figure 2:**
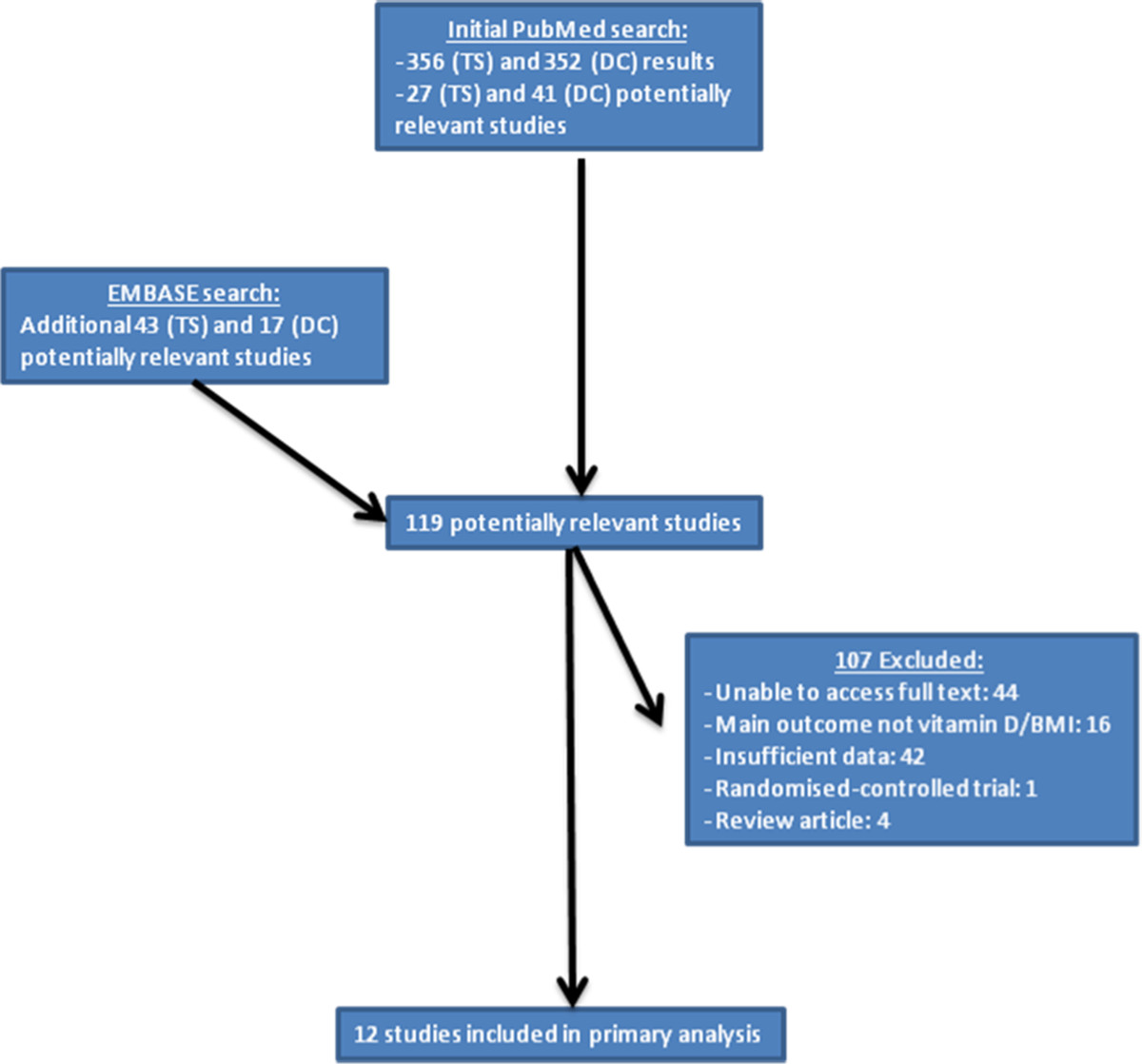
**Flowchart of study selection for the association of obesity and vitamin D.**

All included studies were of epidemiological nature: cohort, case–control, or cross-sectional. Furthermore, all studies included measurements of vitamin D and body mass index (BMI) and assessed the association between the two. We only included those studies with a sufficient power, deemed as including more than twenty cancer cases. Obesity, defined as BMI >30 kg/m^2^, was the main exposure of interest. Low vitamin D levels were the outcome, defined using a cut off of <50 nmol/L, which encompasses both vitamin D insufficiency and deficiency.Initially, titles and abstracts of articles were reviewed by two researchers (Thurkaa Shanmugalingam - TS and Danielle Crawley - DC). If they met initial inclusion criteria both abstract and full text article were reviewed to ascertain whether all inclusion criteria were met. A detailed evaluation of methods and results was undertaken. In the case of any disagreement between the two researchers on article inclusion assessments, the full text article was reviewed by a third researcher (Mieke Van Hemelrijck - MVH). Figure 2 illustrates the study exclusion process.

#### Data extraction

The following details were recorded for each study: author, year of publication, country, type of study, method of vitamin D measurement, statistical tests used, number of subjects with sufficient, insufficient and deficient vitamin D status and BMI of all subjects.

#### Statistical methods

The association between obesity and vitamin D levels was evaluated by calculating the pooled relative risk (RR) with random effects model to allow for possible heterogeneity between studies. Potential publication bias was evaluated using Beggs Test and Eggers funnel plot. All analyses were performed with STATA version 11.0.

### Vitamin D and cancer

A comprehensive literature search of all meta-analyses conducted on the association between vitamin D and cancer was performed. We used computerised search databases (PubMed search followed by an Embase search) to identify full text and abstracts focused on human subjects and published in English language within the last fifteen years. Searches were conducted both with and without MeSH terms for “vitamin D”, “cancer”, “vitamin D receptor”, “polymorphism” and “meta-analysis”. This search was repeated for specific cancer types: “breast”, “colorectal”, “melanoma”, “oesophageal”, “liver”, “lung”, “ovarian”, “endometrial”, “prostate”, “pancreatic” and “kidney” cancer. Moreover, we also searched clinicaltrials.gov for clinical trials focused on “vitamin D supplements” and “cancer” or “neoplasm” [21].

## Results

### Obesity and cancer

Thirty-two meta-analyses were identified from our literature search on obesity and cancer (Table 1). More specifically, all seven meta-analyses on **colorectal cancer** showed a positive association between BMI and colorectal cancer risk [22–28]. When looking at site-specific cancer within colorectal cancer, BMI was only significantly associated with rectal cancer in males. Also **upper gastro-intestinal cancers** (oesophageal, oesophageal gastric junction, gastric and gall bladder cancer) were positively associated with obesity [29–32]. The strongest link was seen for oesophageal cancer with over a two-fold increased risk reported [29, 32]. All four meta-analyses on **liver cancer** reported an increased risk with increasing BMI [33–36], whereas the **lung cancer** meta-analysis reported an inverse association with obesity (RR: 0.79; 95% CI: 0.73-0.85) [20]. Meta-analyses on **pancreatic cancer** reported a positive association with obesity [37–40], which is parallel to the conclusions that can be drawn for **kidney cancer**[41, 42]. For **prostate cancer**[43], a protective effect of obesity was reported for localised disease, whereas obesity was positively associated with metastatic disease [44]. The meta-analysis on **bladder cancer** reported a positive association even when adjustment for smoking was performed [45]. Some variation was observed for **breast cancer** depending on menopausal status and breast cancer subtype [46, 47]. A positive association between obesity and breast cancer was more distinct among postmenopausal women [48]. The meta-analysis on **ovarian cancer** reported a positive association with obesity, with no difference in the histological subtypes of ovarian cancer studied [49]. As for the majority of other cancers [50], there was also a positive association found for **endometrial cancer**[51]. However, this meta-analysis included some studies which used waist circumference as a measure of obesity instead of BMI [51]. The meta-analysis on **melanoma** reported a positive association in men (RR: 1.31; 95% CI: 1.19-1.44), but not in women (RR: 0.99; 95% CI 0.83-1.18) [52].

**Table 1:** **Summary of relative risks from meta-analyses on the association between obesity and risk of cancer**

*Author/Year*	*Pooled RR (95% CI)*	*Number and type of studies included*
**Colorectal**		
Ma Y et al., 2013	1.334 (1.253-1.420)	41 prospective studies
Matsuo K et al., 2012	Per 1 kg/m^2^: 1.03 (1.02-1.04); Males: 1.02 (1.00-1.03); Females: 1.02 (1.00-1.03)	8 cohort studies
Ning Y et al., 2010	Per 5 kg/m^2^: 18% increased risk	56 studies
Harriss DJ et al., 2009	Per 5 kg/m^2^: 1.24 (1.20-1.28)	3 ca/co and 26 cohort studies
Moghaddam AA et al., 2007	1.19 (1.11-1.29)	23 cohort and 8 ca/co studies
Dai Z et al., 2007	Males: 1.37 (1.21-1.56); Females: 1.07 (0.97-1.18)	15 cohort studies
Larsson SC et al., 2007 (*Am J Clin Nutr)*	Per 5 kg/m^2^: Males: 1.30 (1.25-1.35); Females 1.12 (1.07-1.18)	30 prospective studies
**Upper Gastrointestinal**		
Hoyo C et al., 2012	2.39 (1.86-3.06)	12 ca/co studies
Yang P et al., 2009	1.22 (1.06-1.41)	10 cohort studies
Larsson SC et al., 2007 (*Br J Cancer, Vol.96)*	1.66 ( 1.47-1.88)	3 ca/co and 8 cohort studies
Kubo A et al., 2006	Males: 2.40 (1.90-3.20); Females: 2.10 (1.40-3.20)	2 cohort and 12 ca/co studies
**Liver**		
Rui R et al., 2012	1.35 (1.24-1.47)	12 prospective studies
Chen Y et al., 2012	1.83 (1.59-2.11)	26 prospective studies
Larsson SC et al., 2007 (*Br J Cancer, Vol.97)*	1.89 (1.51-2.36)	11 cohort studies
Wang Y et al., 2012	Per 5 kg/m^2^: 1.39 (1.25-1.55)	21 prospective studies
**Lung**		
Yang Y et al., 2013	0.79 (0.73-0.85)	20 cohort and 11 ca/co studies
**Pancreatic**		
Aune D et al., 2012	Per 5 kg/m^2^: 1.10 (1.07-1.14)	23 prospective studies
Genkinger JM et al., 2011	1.30 (1.09-1.56)	14 cohort studies
Jiao L et al., 2010	1.19 (1.05-1.35)	7 prospective cohorts
Berrington de Gonzalez A et al., 2003	1.19 (1.10-1.29)	8 cohort and 6 ca/co studies
**Kidney**		
Mathew A et al., 2009	Per unit BMI: Cohorts: 1.06 (1.05-1.07); ca/co: 1.07 (1.06-1.08)	15 cohort and 13 ca/co studies
Bergström A et al., 2001	Per unit BMI: 1.07 (1.05-1.09)	6 cohort and 22 ca/co studies
**Bladder**		
Qin Q et al., 2013	1.10 (1.06-1.16)	11 cohort studies
**Prostate**		
Discacciati A et al., 2012	Locally advanced per 5kg/m^2^ 0.94 (0.91-0.99); Advanced 1.09 (1.02-1.16)	25 prospective studies
MacInnis RJ et al., 2006	Per 5 kg/m^2^: 1.05 (1.01-1.08)	31 cohort and 25 ca/co studies
**Breast**		
Cheraghi Z et al., 2012	Pre-menopausal: 0.93 (0.86-1.02); Post-menopausal: 1.15 (1.07-1.24)	50 studies
Pierobon M et al., 2013	1.20 (1.03-1.40); Pre-menopausal: 1.43 (1.23-1.65); Post–menopausal: 0.99 (0.79-1.24)	11 ca/co studies
Key TJ et al., 2003	1.36 (1.10-1.85)	8 prospective studies
**Ovarian**		
Olsen CM et al., 2007	1.30 (1.10-1.50)	13 ca/co and 12 cohort studies
**Endometrial**		
Esposito K et al., 2014	2.21 (1.50-3.24)	4 ca/co and 1 cohort studies
**Melanoma**		
Sergentanis TN et al., 2013	Males: 1.31 (1.19-1.44); Females: 0.99 (0.83-1.18)	11 ca/co and 10 cohort studies
**All cancers**		
Renehan AG et al., 2008	Per 5kg/m^2^: Men: Oesophageal: 1.52 (1.33-1.74); Thyroid: 1.33 (1.04-1.70); Colon: 1.24 (1.20-1.28); Renal: 1.24 (1.15-1.34)	141 studies
	Per 5kg/m^2^: Women: Endometrial: 1.59 (1.50-1.68); Gallbladder: 1.59 (1.02-2.47); Oesophageal: 1.51 (1.31-1.74); Renal: 1.34 (1.25-1.43)	

*Abbreviations:* *RR* relative risk, *BMI* body mass index, *ca/co* case–control.

### Obesity and vitamin D

The initial PubMed search produced a total of 356 (TS) and 352 (DC) papers. Further assessment of abstracts and papers based on the above-defined inclusion criteria (Figure 2) resulted in inclusion of 12 studies for primary data analysis (three cohorts, two case–control and seven cross-sectional studies) (Table 2).

**Table 2:** **Summary of studies included in meta-analysis on obesity and vitamin D status**

Author/ Year	Country	Sex	Type of study	Study size
Mai XM et al., 2012	Norway	Both	Cohort	2460 subjects
Goldner WS et al., 2008	USA	Both	Case/control	41 cases/41 controls
Hyppönen E et al., 2006	UK	Both	Cohort	7,198 subjects
Al-Sultan AI et al., 2011	Saudi Arabia	Both	Case/control	76 cases / 84 controls
Campagna AM et al., 2013	USA	Both	Cohort	1,378 subjects
Turer CB et al., 2013	USA	Both	Cross-sectional	12,292 subjects
Guasch A et al., 2012	Spain	Both	Cross-sectional	316 subjects
Poomthavorn P et al., 2012	Thailand	Both	Cross-sectional	179 subjects
Olson ML et al., 2012	USA	Both	Cross-sectional	411 cases/ 87 controls
Shea MK et al., 2011	USA	Both	Cross-sectional	2581 subjects
Elizondo-Montemayor L et al., 2010	Mexico	Both	Cross-sectional	198 subjects
Cizmecioglu FM et al., 2008	Turkey	Both	Cross-sectional	301 subjects

*Abbreviations:* *USA* United States of America, *UK* United Kingdom.

**Figure 3:**
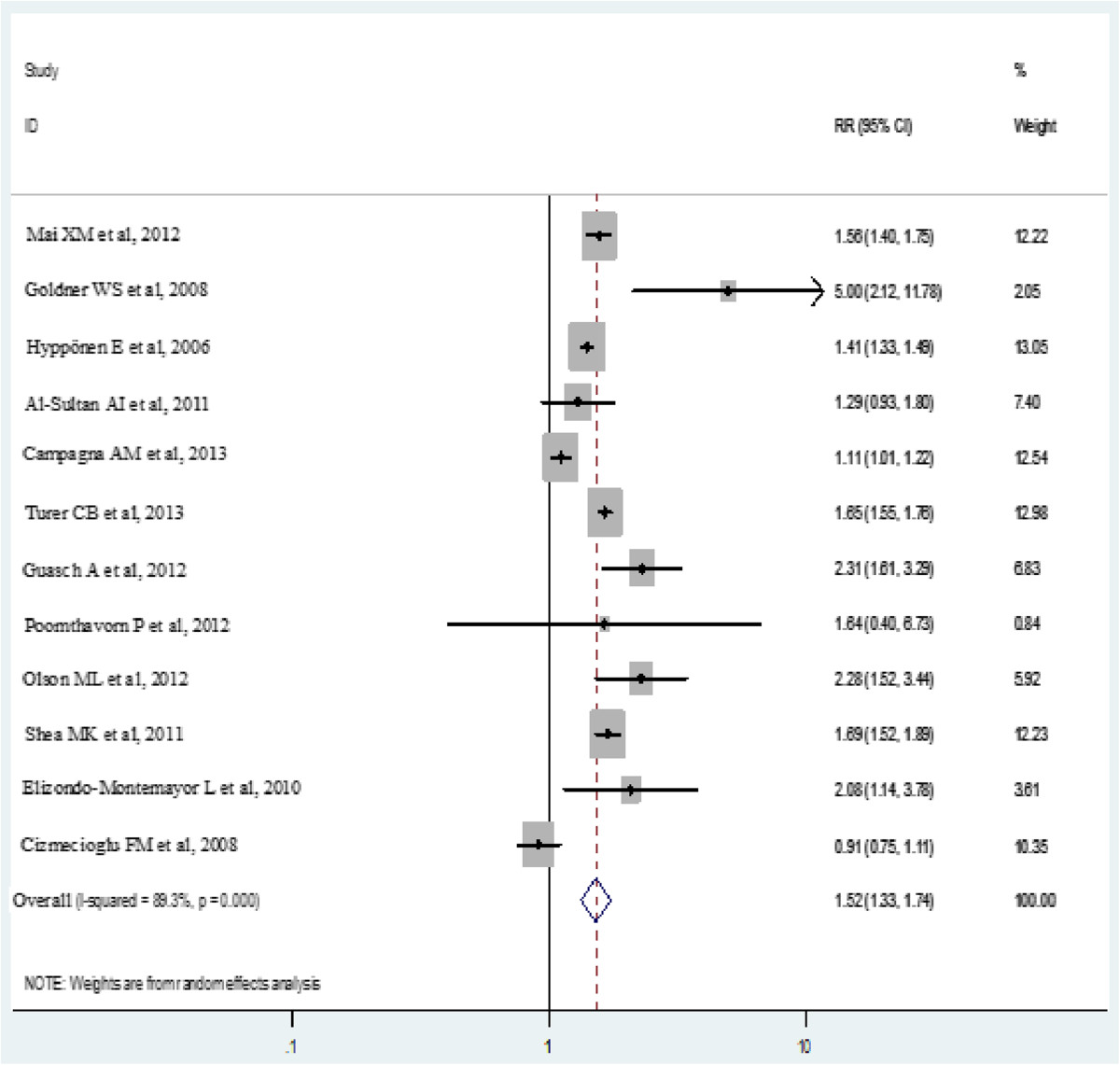
**Forest plot for the association between obesity and low vitamin D levels.**

The random effects analyses showed a pooled relative risk of 1.52 (95% CI: 1.33-1.73) for the association between obesity and low vitamin D status (Figure 3). The I^2^ statistic suggested heterogeneity (I^2^ = 89.4%). There was no difference between those studies looking at children and adolescents combined and those looking at an adult population (RR: 1.52; 95% CI: 1.04-2.26 and 1.53; 95% CI: 1.31-1.80, respectively).

Beggs and Eggers test was used to evaluate publication bias with the funnel plot suggesting the study by Goldner et al. to be an outlier [53] (Results not shown). We performed a sensitivity analysis by excluding this study from our analysis. The pooled estimate of RR did not change drastically, although the link was strengthened to some extent (RR: 1.34; 95% CI: 1.15-1.57).

### Vitamin D and cancer

From the literature search, we identified 21 meta-analyses on the association between circulating vitamin D levels and cancer risk (Table 3), showing different results for different types of cancer. We found 34 clinical trials investigating the effect of vitamin D supplementation on cancer (Table 4) [21]. From these, two studies were terminated, 18 are active, 13 have been completed, and one has an unknown status.

**Table 3:** **Summary of relative risks from meta-analyses on the association between vitamin D status and risk of cancer**

Cancer	Study, publication year	Country	No. of subjects; Type of study	RR (95% CI)	Notes	Measure/Range of vitamin D
**Breast**	Bauer SR et al., 2013	USA	11,656; 9 prospective	0.99 (0.97-1.04)	Pre-menopausal	17-33.1 ng/mL (Mean)
	Bauer SR et al., 2013	USA	11,656; 9 prospective	0.97 (0.93-1.00)	Post-menopausal	17-33.1 ng/mL (Mean)
	Yin L et al., 2010	Germany	Case-control	0.74 (0.69-0.80)		By 20 ng/mL increase
	Chen P et al., 2010	China	11,330; 4 case-control/3 nested case-control	0.55 (0.38-0.80)		Top vs. bottom quantiles (varies)
	Gandini S et al., 2011	France	10 studies	0.89 (0.81-0.98)		By 10 ng/mL increase
	Chen P et al., 2013	China	26,317; 21 studies	0.52 (0.40-0.68)		By 1 ng/mL increase
**Kidney**	Gallicchio L et al., 2010	USA	1,550; 8 cohorts	1.12 (0.79-1.59)	Low <37.5 nmol/L	<37.5 vs. 50-<75 (ref) nmol/L
	Gallicchio L et al., 2010	USA	1,550; 8 cohorts	1.01 (0.65-1.58)	High ≥75 nmol/L	≥75 vs. 50-<75 (ref) nmol/L
**Pancreatic**	Stolzenberg-Solomon RZ et al., 2010	USA	2,285; 8 cohorts	0.96 (0.66-1.40)	Low <25 nmol/L	<25 vs. 50-<75 (ref) nmol/L
	Stolzenberg-Solomon RZ et al., 2010	USA	2,285; 8 cohorts	2.14 (0.93-4.92)	High ≥100 nmol/L	≥100 vs. 50-<75 (ref) nmol/L
**Colorectal**	Touvier M et al., 2011	UK	6 studies	0.96 (0.94-0.97)		200-1,800 IU/L
	Lee JE et al., 2011	USA	8 prospective	0.66 (0.54-0.81)		Top vs. bottom quantiles (varies)
	Ma Y et al., 2011	China	6,715; 9 studies	0.67 (0.54-0.80)		Top vs. bottom categories (varies)
	Yin L et al., 2009 (*Aliment Pharmacol Ther)*	Germany	3,556; 8 studies	0.57 (0.43-0.76)		By 20 ng/mL increase
	Gorham ED et al., 2007	USA	1,448; 5 nested case–control	0.49 (0.35-0.68)		Top vs. bottom quintile (varies)
	Gandini S et al., 2011	France	9 studies	0.85 (0.79-0.91)		By 10 ng/mL increase
**Prostate**	Gilbert R et al., 2011	UK	14 cohort/nested case–control	1.04 (0.99-1.10)		By 10 ng/mL increase
	Yin L et al., 2009 (*Cancer Epidemiol)*	Germany	7,806; 11 studies	1.03 (0.96-1.11)		By 10 ng/mL increase
	Gandini S et al., 2011	France	11 studies	0.99 (0.95-1.03)		By 10 ng/mL increase
**Ovarian**	Yin L et al., 2011	Germany	2,488; 10 longitudinal	0.83 (0.63-1.08)		By 20 ng/mL increase
**All Cancers**	Yin L et al., 2013	Germany	5 studies	0.89 (0.81-0.97)	Total cancer incidence	Per 50nmol/L increase
			13 studies	0.83 (0.71-0.96)	Total cancer mortality	Per 50nmol/L increase
			3 studies	0.76 (0.60-0.98)	Total cancer mortality (women)	Per 50nmol/L increase
			5 studies	0.92 (0.65-1.32)	Total cancer mortality (men)	Per 50nmol/L increase

*Abbreviations:* *RR* relative risk, *USA* United States of America, *UK* United Kingdom, *ref* reference.

**Table 4:** **Summary of clinical trials on vitamin D status and cancer risk**

Cancer	NCT#	Country	No. of subjects	Intervention	Status	Main finding
Colorectal	NCT00870961	USA	22	VDS	Terminated	Not reported
Colon	NCT00470353	USA	8	VDS, CaCO_3_	Terminated	Not reported
Lung	NCT01631526	Canada	80	VDS	Active recruitment	
Colorectal	NCT01074216	USA	49	VDS	Active, not recruiting	
Not specified	NCT01169259	USA	20,000	VDS, fish oil	Active recruitment	
Not specified	NCT01463813	Finland	18,000	VDS	Active recruitment	
Prostate	NCT0887432	USA	100	VDS	Active recruitment	
Colorectal	NCT01516216	USA	120	VDS, folfox, bevacizumab	Active recruitment	
Ovarian	NCT01744821	USA	80	VDS	Active recruitment	
Breast	NCT01224678	USA	180	VDS	Active, not recruiting	
Colon, prostate	NCT00585637	USA	328	VDS	Active, not recruiting	
Lymphoma, leukaemia, colon, breast, rectal	NCT01787409	USA	956	VDS	Active, not recruiting	
Breast	NCT01747720	Canada	376	VDS	Active recruitment	
Breast, leukaemia, colon, lymphoma, lung, myeloma	NCT01052051	USA	2,300	VDS	Active, not recruiting	
Prostate	NCT01325311	USA	50	VDS, genistein	Active recruitment	
Prostate	NCT00022412	USA	60	VDS	Active, not recruiting	
Breast	NCT01097278	USA	200	VDS	Active recruitment	
Breast	NCT01816555	USA	20	VDS	Active recruitment	
Leukaemia	NCT01521936	USA	4	VDS	Active, not recruiting	
Solid tumours	NCT00004926	USA	NA	VDS	Completed	Not reported
Leukaemia, myeloma	NCT00068276	USA	NA	VDS	Completed	Not reported
Prostate	NCT00004928	USA	NA	VDS, zoledronate	Completed	Not reported
Colorectal	NCT01574027	USA	55	VDS	Completed	Not reported
Breast	NCT01240213	USA	218	VDS	Completed	Not reported
Colorectal	NCT00208793	USA	92	VDS	Completed	Not reported
Colon	NCT00298545	USA	10	VDS, calcium	Completed	Not reported
Prostate	NCT01045108	USA	52	VDS	Completed	Not reported
Prostate	NCT00524680	USA	128	VDS	Completed	Not reported
Pancreatic	NCT00238199	USA	25	VDS, docetaxel	Completed	Not reported
Prostate	NCT00004043	USA	25	VDS	Completed	Not reported
Breast, colon	NCT00000611	USA	18,176	VDS, CaCO_3_	Completed	No effect
Not specified	NCT00352170	USA	1,179	VDS, CaCO_3_	Completed	All-cancer risk reduction
Colorectal	NCT01150877	Canada	40	VDS	Unknown	
Prostate	NCT00741364	Canada	90	VDS	Unknown	
Prostate	NCT00482157	USA	24	VDS	Unknown	
Colorectal	NCT01403103	NA	0	VDS	Withdrawn	

*Abbreviations:* *NCT#* national clinical trial number, *USA* United States of America, *VDS* vitamin D supplement, *CaCO*_*3*_ calcium carbonate, *NA* not applicable.

All six meta-analyses on **colorectal cancer** reported that circulating vitamin D levels were inversely associated with cancer risk [54–59]. A pooled analysis from multiple cohort studies on **pancreatic cancer**, suggested no significant association for participants with low vitamin D levels. Those with vitamin D levels ≥100 mmol/L were at a statistically significant twofold increase in pancreatic cancer compared to those with normal vitamin D levels [60]. The pooled analysis for **kidney cancer** only found a statistically significant decreased cancer risk among women when vitamin D levels was ≥75 nmol/L [61]. In contrast, all three meta-analyses on **prostate cancer** found no evidence for an inverse association with vitamin D levels [58, 62, 63]. Results from four out of five meta-analyses showed an inverse association for **breast cancer**, with the highest quartile of vitamin D levels decreasing the risk of breast cancer [58, 64–67] compared to the lowest quartile. However, it is interesting to note that case–control studies generally report an inverse association, whereas nested case control studies reported null-findings [58, 64–67]. The meta-analysis on **ovarian cancer** reported a non-statistically significant inverse association with high serum vitamin D levels [68]. Finally, the meta-analysis on **total cancer incidence and mortality** reports a moderate inverse relationship with circulating vitamin D concentrations [69].

From the 13 completed **clinical trials** evaluating the effect of vitamin D supplementation on cancer risk, only two have reported results [70, 71]. One randomised trial focused on risk of colorectal cancer over a period of seven years in a double-blinded, placebo-controlled setting, where one group of postmenopausal women received calcium and vitamin D3 supplementation and the other group received placebo [70]. The study found no statistically significant effects of calcium or vitamin D3 supplementation on the incidence of colorectal cancer. The other completed trial had a similar design, but focused on risk of all cancers in postmenopausal woman receiving 1400–1500 mg supplemental calcium/d alone, supplemental calcium plus 1100 IU vitamin D3/d, or placebo during a follow-up of four years [71]. In contrast, this trial found that those women on vitamin D supplementation had a lower risk of cancer, compared to the placebo group when the analysis was confined to cancers diagnosed after the first 12 months (RR: 0.23; 95% CI: 0.09-0.60). No statistical analyses were performed for specific types of cancer [71].

## Discussion

To date no mediation analyses have been performed for the effect of obesity on cancer risk through vitamin D. Even though we could not find the question addressed in one single study, it is still of interest to discuss study design and methodology of studies published on any of the three questions, (Figure 1) to interpret the validity of a potential mediation effect of vitamin D [72].

### Obesity and cancer

The majority of meta-analyses included in our review reported positive associations between obesity and risk of cancer, showing that the strength of this association varies between cancer sites, sex, and in breast cancer, the menopausal status. The World Cancer Research Fund (WCRF) suggests that obesity is associated with increased risk of oesophageal adenocarcinoma, pancreatic, colorectal, postmenopausal breast, endometrial and renal cancer [73].

There are several molecular mechanisms suggested to explain the increased risk of cancer in obese people. The most commonly postulated being the “insulin–cancer hypothesis” [74], suggesting that obesity results in chronic hyperinsulinaemia. Prolonged hyperinsulinaemia leads to raised insulin like growth factor 1 (IGF-1) levels, which are known to produce cellular changes leading to carcinogenesis via increased mitosis and reduced apoptosis. Secondly, in hormonally-driven cancers, such as endometrial and post-menopausal breast cancer, the increased risk may be partly explained by an increase in circulating levels of sex steroid hormones. In the post-menopausal state, the majority of oestrogen is derived from adipose tissue rather than from the ovaries, potentially explaining the discrepancy between pre- and post-menopausal women. Thirdly, obesity is thought to result in a state of chronic inflammation and this has an effect on the cytokine microenvironment. These changes lead to an increase in tumour cell motility, invasion and metastasis. The change in the cytokine milieu has been suggested as a possible mechanism in several cancers including post-menopausal breast cancer [75].

The majority of the meta-analyses in our literature review included a substantial number of studies, with consistent study design. However, the meta-analysis on endometrial cancer [51] only included five studies of which some used other markers than BMI to define obesity (i.e. waist circumference). None of the studies to date included additional information on vitamin D status.

In summary, there is consistent accumulating evidence for an association between obesity and risk of certain cancer with several suggested molecular mechanisms that can potentially explain these raised risks. However, the role of vitamin D is not addressed in detail in these studies.

### Obesity and vitamin D

To our knowledge this is the largest meta-analysis to date on the association between circulating vitamin D levels and obesity. The pooled estimates suggest an inverse relationship between vitamin D and obesity.

The possible relationship between vitamin D and obesity was firstly described by Rosenstreich et al. in 1971 [76], who suggested that adipose tissue serves as a large storage site for vitamin D to protect against toxicity from vitamin overdose. The inverse association between obesity and vitamin D is thus thought to be a result of increased metabolic clearance in adipose tissue [77]. However, it has recently been suggested that this association is more complex since bariatric surgery solely has temporary effect on improving circulating vitamin D levels [78]. It is also postulated that obese individuals are less likely to engage in outdoor physical activity and dress differently than non-obese individuals, hence leading to decreased sun exposure [79, 80]. Wortsman et al. have shown that the bioavailability of cutaneously synthesised vitamin D decreases by >50% in obese people [81]. Even though exposure to sunlight is the main source of vitamin D synthesis [82, 83], its ultraviolet radiation is also known to increase risk of developing malignant melanoma of the skin [83]. In general, epidemiological studies have described that the highest incidence of melanoma is seen in fair-skinned population living closest to the equator [82, 84]. Within this population the highest risk is seen in those who report sunburn or intermittent sun exposure [85–87]. Furthermore, Newton-Bishop et al. found that low vitamin D levels were associated with a thicker and more aggressive melanoma, with a poorer outcome [88]. Overall, vitamin D levels are known to be lower in obese individuals and several studies have observed that increased BMI is associated with an increased risk of developing melanoma [89–91]. However, to date it has not been clarified whether the risk of melanoma in obese individuals is due to lower vitamin D levels associated with high BMI or less sun exposure.

Furthermore, certain vitamin D receptor (VDR) polymorphisms are associated with obesity [92, 93]. Upon ligation with calcitriol, the VDR couples with the retinoid X receptor (RXR) forming the VDR/RXR complex. This complex then further recruits other molecules, and finally binds to vitamin D response elements in the nucleus to activate the transcription of vitamin D target genes [92, 93]. Preclinical studies report expression of human VDR in mature mice adipocytes. This results in increased adipose mass and decreased energy expenditure [94] and expression of VDR in preadipocyte cell lines; this inhibits adipocyte differentiation [95]. A positive association between obesity and the Taq1 gene was also reported in a Greek case–control study [96].

In contrast, some suggest that low vitamin D itself promotes obesity. Kong and Li demonstrated that vitamin D levels may block the expression of downstream adipocyte components such as fatty acid synthase, which consequently suppresses adipogenesis [97]. One interventional study investigated the effects of vitamin D on weight loss and showed that those with higher baseline vitamin D experienced a greater degree of weight loss than those with lower baseline vitamin D [98].

In conclusion, our meta-analysis reports a modest inverse association between obesity and low vitamin D levels. The underlying biological mechanisms are unknown. The majority of studies point towards the hypothesis that, vitamin D stored in fat tissue increases local vitamin D concentrations causing activation of the VDR in adipocytes. This may lead to low energy usage and further promotion of obesity [94].

### Vitamin D and cancer

In this literature review only those meta-analyses focusing on colorectal cancer found a consistent inverse association between circulating vitamin D levels and cancer risk [54–59]. In contrast, of the two completed clinical trials for which results are published to date, one showed no effect on colorectal cancer risk and one showed a protective effect for all cancer risk [70, 71].

A protective effect of vitamin D in colorectal cancer was first reported by Garland and Garland [99]. Despite the inconsistency in epidemiological findings [54–61, 64–68], there is preclinical evidence linking vitamin D and cancer, suggesting that vitamin D has anti-proliferative effects via mechanisms such as G0/G1 arrest, differentiation, and induction of apoptosis [100].

More specifically, it is suggested that vitamin D has anti-tumour effects through its binding with the VDR. Several animal and cell culture models showed that VDR plays a key role in the anticancer effects of circulating vitamin D [9–11]. For instance, it has been reported that downregulation of VDR correlates with poor prognosis in colon cancer [101], suggesting that some of the discrepancy observed in epidemiological studies can be explained through gene polymorphisms [102]. VDR polymorphisms have been associated, both positively and inversely, with risk of cancer depending on the type of cancer, polymorphism, and other factors such as sun exposure or circulating vitamin D levels [8, 103]. For instance, a meta-analysis for prostate cancer found no association between the recessive genotype and the risk of prostate cancer relative to the dominant genotype of Fok1 [104]. To date, the importance of the role of VDR polymorphisms in carcinogenesis is unclear [101], but when analysed with additional factors like VDR haplotype combinations, vitamin D serum levels and other confounders, polymorphisms have been shown to play an important factor in cancer prognosis [105–107].

Interestingly, several parts of the immune system (i.e. macrophages, neutrophils, or natural killer cells) also express the VDR, but the related effects remain to be elucidated [12]. It has for instance been suggested that vitamin D can weaken antigen presentation by dendritic cells, which results in suppression of their capacity to activate T cells. Furthermore, activation of the VDR promotes a shift towards T helper 2 responses, leading to antibody-mediated immunity and promoting a chronic state of disease [108, 109]. Hence, it is plausible that vitamin D has an immunosuppressive effect, which leaves tumour cells without the necessary immunosurveillance to stop them from proliferating. Thus, this suggests that the above-described potential anti-cancer effect of vitamin D most likely occurs through different mechanisms than the immune system. Most literature to date on vitamin D and the immune system has focused on autoimmune and infectious diseases, with scarce literature focusing on cancer.

In 2008, the International Agency for Research on Cancer concluded that evidence for an association between vitamin D and cancer was inconclusive, and highlighted the need for a clinical trial with specific focus on vitamin D and colorectal cancer [101]. The inconsistent findings from two trials for which results are published to date [70, 71] may be explained by the lower dose of vitamin D in the first study (i.e. 400 IU vs. 1100 IU). Furthermore, baseline vitamin D levels were lower in the second trial (i.e. 42 nmol/L vs. 71.8 nmol/L). Thus, despite the large amount of preclinical studies trying to establish a link between vitamin D and cancer, the contradictive findings from large epidemiological studies indicate that it is prudent to wait for more results from the 34 currently on-going trials to draw a reliable conclusion.

### Is vitamin D a mediator for the association between obesity and cancer?

When assessing the three conditions required for vitamin D to be a mediator we found only partial fulfilment [110]. The literature shows consistent evidence for an association between vitamin D and obesity. However, there was lack of studies showing a consistent link between vitamin D and cancer after adjustment for obesity. To date, only two clinical trials have published their results with inconsistent findings. Furthermore, to our knowledge no study has assessed the mediation effect of vitamin D by quantifying the extent of obesity on cancer, which could be explained by a potential mediator.

Several other difficulties occur when assessing the mediation effect of vitamin D in the context of obesity and cancer. Dichotomisation of vitamin D exposure (low versus normal) could lead to misclassification in exposure levels. Those with extreme high values of vitamin D may have been included in the “normal” group. Hence, bias can occur when there is misclassification of the mediator [13]. Studies to date have used different cut-offs to define vitamin D deficiency, which can potentially be addressed with a dose–response assessment of this mediator. Unfortunately, it was not possible in this meta-analysis to use dose–response data [111] as the number of relevant studies available to date was small, and the qualitative classifications of circulating vitamin D levels varied. Furthermore, the effects of dietary supplements on circulating vitamin D levels needs to be accounted for, and very few studies took this into account [112]. The latter does not necessarily affect blood levels of vitamin D, but it may influence the biological role of vitamin D. Within-person variation may also affect the results of our meta-analysis, as only one measurement in time might not be representative of a person’s average vitamin D level. Moreover, it is important to address potential important confounders for the different associations studied [13, 72]. For instance, when evaluating the effect of the mediator (vitamin D) on the outcome (cancer), one has to consider age, sex, use of dietary supplements, ethnic variations, calcium intake and sun exposure [113], as they may be effect modifiers for the association between obesity and vitamin D. It has been argued that it is also important to address the strength of the association between these mediator-exposure confounders and both the exposure (obesity) and the outcome (cancer) [13]. With respect to the mediation effect of vitamin D, one also needs to evaluate whether there is a potential interaction affecting the link between the exposure (obesity) and the mediator (vitamin D) [13]. Effect modification may also have an effect on the link between the mediator (vitamin D) and the outcome (cancer), as is suggested by the different polymorphisms affecting the VDR [8].

Additionally, the current systematic literature reviews are prone to the heterogeneity related to observational studies. For example, for the studies focused on vitamin D and obesity the included studies recruited adults residing in a particular town [114], from medical centres [115–117], from sample surveys [2, 118], and those undergoing bariatric surgery [53]. Children were recruited from schools [119, 120], hospitals [121, 122], and sample surveys [123]. Vitamin D levels were measured using either an immunoassay [2, 53, 114, 115, 118–121, 123] or a high-performance liquid chromatography [116, 122]. Anthropometric data, including weight, height, waist circumference and BMI, were recorded for all participants [119, 120, 122]. Furthermore, information on dietary, physical activity and sun exposure were collected either by parental report during in-person interviews [123], and interview-administered questionnaires [114, 122]. These questionnaires may be subject to recall bias, as participants may not always give accurate data [124, 125] due to the time interval, degree of detail, personal characteristics, significance of events, social desirability or interviewing techniques [126]. Furthermore, despite proven validation, many food questionnaires have been found to be imprecise [127], due to the fact that people tend to answer these type of questions based on what their dietary routines are, more than on the real consumption. These memories are usually influenced by sex, age, and concerns about weight or body image [128].

A strength of these systematic reviews and meta-analysis is that we made all possible efforts to include all relevant publications available to date through various sources, including grey literature, and the two main online databases (PubMed and Embase). In addition, clearly defined objective criteria for exposure, outcome, and other study characteristics were specified *a priori*.

## Conclusion

To understand how vitamin D may play a role in the association between obesity and carcinogenesis, we assessed the strength of these three associations: 1) There was a consistent positive association between obesity and cancer with relative risks varying between 1.10 and 1.90 when addressing the existing literature; (2) Our new meta-analysis illustrated an association as strong as 1.50 between obesity and low vitamin D levels; (3) The literature for vitamin D status and cancer risk only showed consistent evidence for an inverse association with colorectal cancer. From these reviews, it seems that the significance of the mediating role of vitamin D in the biological pathways linking obesity and cancer is low. This review emphasises that further research specifically addressing the relationship between obesity, vitamin D and cancer risk in one study is needed.

## Authors’ original submitted files for images

Below are the links to the authors’ original submitted files for images. Authors’ original file for figure 1 Authors’ original file for figure 2 Authors’ original file for figure 3

## Abbreviations

VDR: Vitamin D receptor
BMI: Body mass index
RR: Relative risk
WCRF: World cancer research fund
IGF-1: Insulin like growth factor 1
RXR: Retinoid X receptor
ca/co: Case–control.
