# Supplementary figures and images for: Obesity and cancer: the role of vitamin D
Source: BMC Cancer. 2014 Sep 25;14:712. doi: 10.1186/1471-2407-14-712 (PMC4182855; doi:10.1186/1471-2407-14-712)

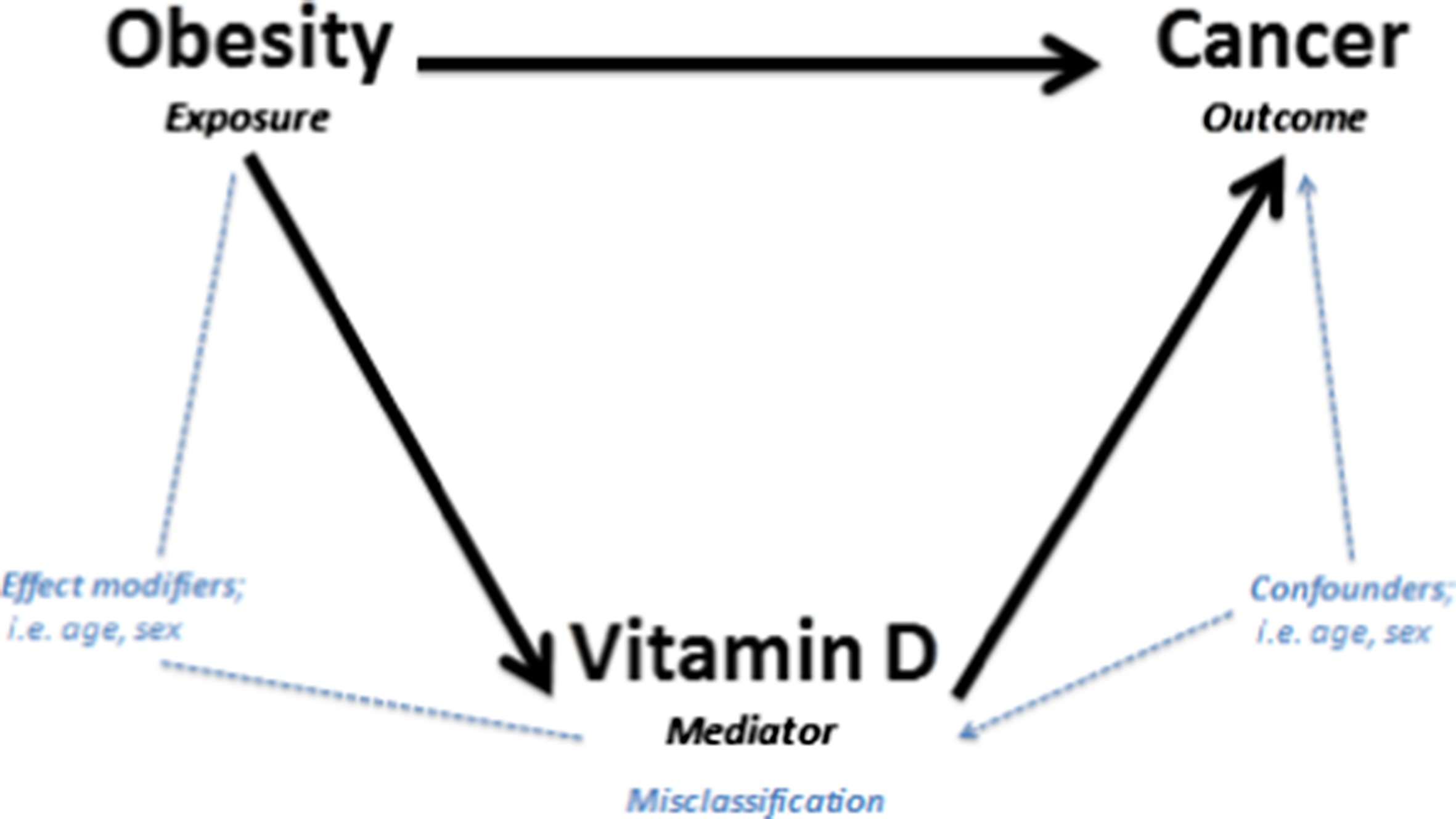

Supplement: Supplementary file 1 — Authors’ original file for figure 1 [file 12885_2014_4886_MOESM1_ESM.tif]

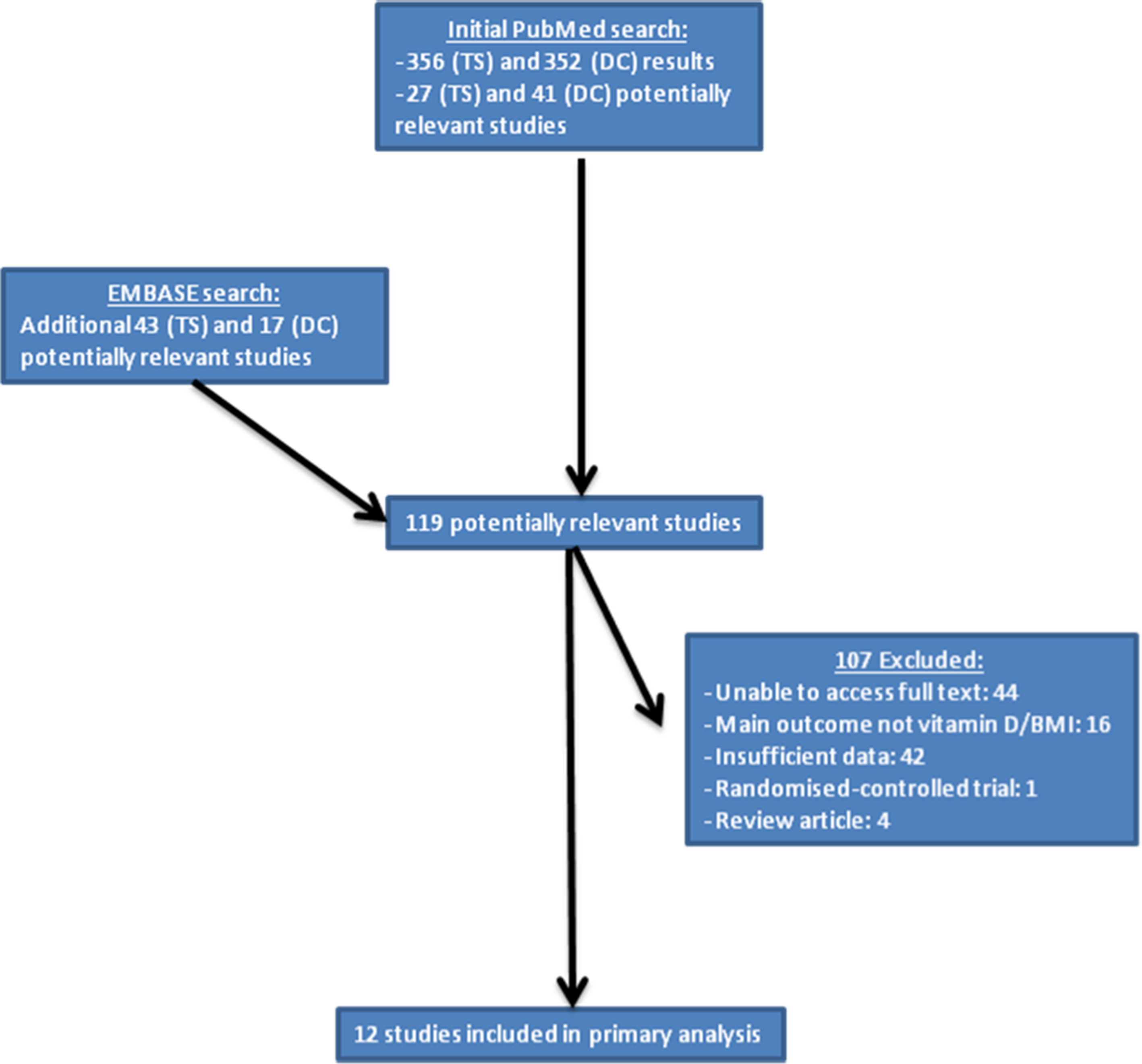

Supplement: Supplementary file 2 — Authors’ original file for figure 2 [file 12885_2014_4886_MOESM2_ESM.tiff]

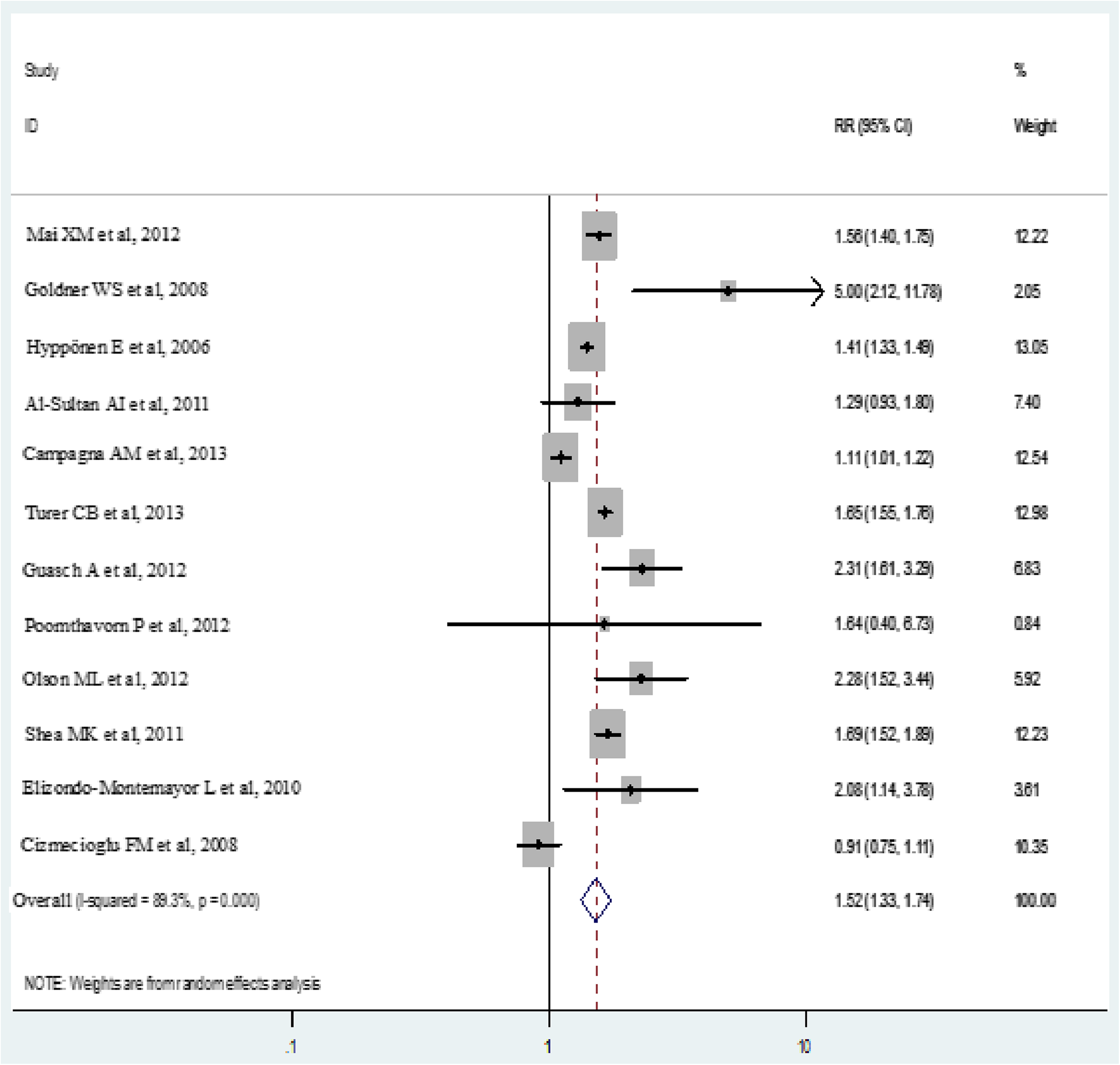

Supplement: Supplementary file 3 — Authors’ original file for figure 3 [file 12885_2014_4886_MOESM3_ESM.tiff]
